# Consumption-Related Health Education Inequality in COVID-19: A Cross-Sectional Study in China

**DOI:** 10.3389/fpubh.2022.810488

**Published:** 2022-04-25

**Authors:** Jialu You, Jinhua Zhang, Ze Li

**Affiliations:** ^1^Shanghai University of Finance and Economics, Shanghai, China; ^2^Yunlin University of Science and Technology, Douliu, China; ^3^Hubei University of Technology, Wuhan, China

**Keywords:** health inequalites, machine learning regressors, health education, household consumption, income-expenditure theory

## Abstract

**Background:**

The COVID-19 pandemic influences various aspects of society, especially for people with low socioeconomic status. Health education has been proven to be a critical strategy in preventing a pandemic. However, socioeconomic characteristics may limit health education among low socioeconomic status groups. This study explores consumption-related health education inequality and the factors that contribute to this, which are variable across China during COVID-19.

**Methods:**

The 2020 China COVID-19 Survey is a cross-sectional study in China, based on an anonymous online survey from 7,715 samples in 85 cities. It employed machine-learning methods to assess household consumption and other contributing variates associated with health education during the pandemic. Concentration Index (CI) and Horizontal Index (HI) were used to measure consumption-related inequalities in health education, respectively. Moreover, Wagstaff decomposition analysis was employed to identify other contributing variables to health education inequality.

**Results:**

The result indicates that participants with more education, better income, and positive consumption preferences undertake higher health education during COVID-19. The CI and HI of consumption-health education inequality are 0.0321 (*P* < 0.001) and 0.0416 (*p* < 0.001), respectively, which indicates that health education is concentrated in wealthy groups. We adapted Lasso regression to solve issues and omit variables. In terms of other socioeconomic characteristics, Annual Income was also a major contributor to health education inequalities, accounting for 27.1% (*P* < 0.001). The empirical results also suggests that education, health status, identification residence, and medical health insurance contribute to health education inequality.

**Conclusions:**

The difference in Household consumption, annual income, rural and urban disparity, and private healthcare insurance are critical drivers of health education inequality. The government should pay more attention to promoting health education, and healthcare subside policy among vulnerable people. Significantly to improve awareness of undertaking health education with lower education, rural residential, to enhance confidence in economic recovery and life after COVID-19.

## Background

Since the COVID-19 outbreak, it has spread all over the world with rising fatality cases (1). As of October 28, 2021, confirmed cases of the novel coronavirus disease (COVID-19) exceeded 246 million, with ~498 million deaths worldwide (World Wide, 2021). Due to COVID-19 being highly contagious and there being a high mortality rate, preventive measures and physical distance have been adopted to decrease rates of transmission. The current pandemic profoundly influences population health and the economies across the world (2). The unprecedented COVID-19 pandemic brought to the fore the important role that health education plays in securing and preventing cases in individuals. However, the pandemic also highlights the health education inequalities of vulnerable groups (3).

The government of China first began healthcare system reform in 2007, with the World Health Organization (WTO) providing reform proposals. The healthcare system includes two aspects: Urban Residents Medical Healthcare System (URBMHI), and the New Type Rural Cooperative Medical Healthcare System (NRCMHS). China has had three main healthcare reforms between 2007 and 2020, firstly, expanding and sustaining 95% coverage of the basic healthcare insurance system until 2019. Secondly, the per capita healthcare premium was increased from 100 to 700 RMB, with a percentage of 70% of public subsidies for urban resident's medical healthcare system. In 2020, the government subsidy for resident medical security is 550 RMB per person; finally, expanding healthcare coverage of reimbursement for basic drugs. For example, the cost of the hepatitis B antiviral drugs, tenofovir disoproxil fumarate (TDF), and entecavir drugs were decreased from 9,000 to 70 RMB. The healthcare system reforms relieve individual concerns about illness and healthcare, however, there is still inequality between urban and rural populations. For example, the new-rural medical healthcare system mainly focuses on township hospitals, and city hospitals receive relatively less attention. Moreover, the average reimbursement with rural residential in city hospitals is lower than 20 percent than urban residential. This is particularly the for health infrastructure, health education, and resource inequality among healthcare systems, which are still areas of high concern during the COVID-19 pandemic.

Therefore, this study measures the extent of health education inequalities in healthcare systems using representative, cross-sectional household survey data during the pandemic. It also evaluates the relationship between health education inequality and household consumption to improve welfare for those on lower incomes during the COVID-19 pandemic.

## Literatures

### Health Inequality Theory

Health inequality refers to whether there are observed differences experienced by an individual or groups that are unfair (4). The main explanation for health disparities points to material factors, including income, consumption, food, and other materials resources (5). An alternative explanation of health disparities points to psychology social material factors, including racial, ethnic, education, and psychological reactions to social experiences (6).

Most studies to date have explored income-related health material inequality and income-related health psychology social inequality (7, 8). The *absolute income hypothesis* is the most common theory explored, which implies that an individual's health only depends on their income (9); however, the absolute income hypothesis ignores the fact that with economic development, the material goods needed to fully participate in society also become wealthier. As a result, those with a static income may fall behind in a changing society, potentially suffering health education or material inequality from being unable to keep up with average social change standards (10). Another theory is the relative income hypothesis, which outlines that individuals compare their income to that of others, considering relative income through social reputation, residential location, other mechanism effects, and health education (11). Relative income and absolute income do not represent the life course perspective because income changes over time (12). Consumption is more representative of the life course perspective: implying that consumption is determined by life cycle expectancy income, based on life cycle theory (13).

### Consumption Related to Health Education Inequality

Little evidence exists on the role of consumption and how it relates to health education inequality. Some studies have investigated the association between consumption and health status (14, 15). These findings suggest that consumption preference and consumption structure have significant positive effects on health inequality. A study by Wagstaff (16) supports this relationship, examining socioeconomic inequality in health across 19 countries, indicating that spending index (household consumption) is the main factor of socioeconomic inequality in health.

The above studies suggest that consumption influences socioeconomic inequality and health inequality, but evidence on health education inequality remains scarce. Health Educational inequalities are among the most consistent findings in social epidemiological literature.

In China, the government have controlled the pandemic by spreading educational materials on preventing COVID-19, including instructions about regular hand washing and wearing face masks. However, the success of these measures does not depend on individual health education (17). Inequality in health education may further exacerbate economic outcomes and the healthcare gap in developing countries. Evidence shows that health education is essential in tackling pandemics (18). Health education about the coronavirus can improve individuals from undertaking certain behaviors and help with mental health (19). In brief, equitable health education comprises a key area in today's health system. However, considering the cost of health information, inequality in health education is associated with economic outcomes (20). Individuals with limited health education may struggle in less wealthy healthcare services and panic mentally during pandemics (21).

We first used the cross-sectional data during COVID-19 to test the relationship between household consumption and health education inequality. We then used machine learning to measure the impact of household consumption on health education inequality, providing further human capital reference information and policy implications different from the existing literature.

## Materials and Methods

### Data Source and Sample

This cross-sectional study was drawn from the general population of China from March 2020 to May 2020. Accounting for the social distance, the data was collected through WeChat (China‘s leading messaging and social networking mobile application, with monthly active users exceeding one billion since 2018). To achieve the sample objective and representative statistics, we recruited diverse national samples through snowball sampling and stratified multistage clusters across China (22). The final survey includes 7,715 households over 18 years old, covering 301 communities in 85 cities, excluding the city most severely affected during the pandemic, Wuhan. The survey reliability and validity analysis results had a 99% confidence level and 3% marginal measurement error.^1^ Accounting for the data that is nationally representative, the data was sorted into weighted strata, with each stratum-weighted measurement requiring a minimum of 30 participants, following Lazarus (23).

### Survey and Data Analysis

A questionnaire was designed based on literature and the National Health Department (24, 25) but some questions were modified to the appropriate purpose of this study. The questionnaire was conducted in the Chinese language, and the interviewee was informed of the survey's objectives. The interviewee was free to withdraw at any time, and the process was anonymous and confidential.

We collected data on household consumption and contributing variables to health education during COVID-19 on the survey questionnaires, which contained four parts, 15 items. The four parts include individual socio-economic characteristics, community socio-economic characteristics, household consumption characteristics, and health education, respectively.

We classified individual characteristics into three groups using the definition described by the China Health and Retirement Longitudinal Study (CHRLS),^2^ which is comprised of individual demographic characteristics (age; gender; marital state; birthday; education year; education level; Ethnicity; and Hukou Registration), individual social characteristics (Party Member; Leader; Occupation types; Occupation sites; family social reputation), and individual economic characteristics (year income; total income; income structure; residence type; Fix Assets with household).

The community socio-economic characteristics being divided into two groups based on the definition in CHRLS, which contains two items, community social characteristics and economic characteristics, respectively. It includes geography features; location; duration with the community; community scale (number of households); and community leader's individual characteristics. In terms of economic characteristics, including healthcare service; health infrastructure; health activity; community hospital; government funds.

We followed the Wagstaff (16) spending index and CHRLS datasets to measure household consumption, comprising: consumption habit; consumption preference; consumption instruments; consumption location; and consumption change after COVID-19. Consumption habit contains subsistence expenditure; development expenditure; enjoying expenditure. The consumption preferences included alcohol, smoking, green foods, healthcare drugs, and commercial healthcare insurance. The consumption instruments denote cash, phone or credit card, and consumption location including shopping online or motor stores. Furthermore, we compared consumption changes after COVID-19 in relation to consumption behaviors.

All responders completed the questionnaire after consent to participate in the survey. Each answer ranged from “totally agree,” “agree,” “normal,” “disagree,” or “totally disagree.” and was evaluated by a five-point Likert scale.

To construct a diversity variable to measure health education, we calculated health education by assignment values of the four dimensions of health education (26), which included data on participants: community health education; COVID prevention health education; a self-psychology report toward COVID-19; and infectious illness health education (HIV; Shigella; HBV). All answers ranged from 1 to 8 score (s). A score of 1 was considered to have a lower level, and the score of 8 was a high level and the full score was 32 points. Considering regression easily, we take the logarithm of health education scores.

## Measurements

### Independent Variable

Based on a four part survey, we constructed health education variables. Respondents were asked to respond to four items of health education, including participant's community health education, prevent health education about COVID-19, a self-psychology report toward COVID-19, and infectious illness health education (HIV; Shigella; HBV), respectively. The score of each item ranged from one to eight. Uncertain (do not know) answers were given a score of zero, with eight scores indicating better health education levels per item. We generated a health education score by summing the values for all four responses, which yielded a total health education score of between 0 and 32, with higher values indicating better health education.

### Dependent Variable

The independent variables of household consumption were assessed by five items. Each question was calculated using a 5-points scale “1 = totally agree,” “2 = agree,” “3 = normal,” “4 = disagree,” “5 = totally disagree.” To construct lasso regression to explore the relationship between health education and household consumption, we based on the Principal component analysis (PCA) to analyze five dimensions of household consumption. Principal component analysis can accurately reflect the level of household consumption with the weighting factor regression (27), and the internal reliability values represented by Cronbach's α, the values equal 0.85, indicating internal reliability. To facilitate the regression, all scores are standardized in the range from 0 to 1. The household consumption attitudes with a close 1 score, indicate households with higher consumption.

### Control Variables

The control variables included two aspects of individual characteristics and community characteristics, respectively. For example, individual characteristics include age square, gender, marital status (unmarried, married); education years (below 6 years = “elementary school or below;” 9 years = junior school; 12 years = high school diploma; 16 years = bachelor's degree; upper 16 years = master's degree or above); monthly income; annual income; health insurance coverage, health report, and residence regional (rural, town, or city). The annual income is used as the index to evaluate the level of health education inequality. The age variable is from 18 to 80. The gender of participants was assigned a value of 1 if the respondent was male and 0 if female. Marital status is also represented as a binary variable, with 1 representing being married and 0 otherwise.

Health insurance was also treated similarly to Gender and Marital status. The self-health psychology report was divided into five categories: naughty, nasty, normal, well, and very well. Monthly income was grouped based on the quantiles income, divided into lower income (<2,000 RMB), medium income (range from 2,000 to 5,000 RMB), and higher income (upper 5,000 RMB). Considering the measurement error caused by the omitted control variable, we used Lasso machine regression to explore the relationship between health education and household consumption (28). All regression analyses were conducted using STATA and R software.

## Statistical Analysis

### Household Consumption and Health Education

To analyze the relationship between health education and household consumption, we based on the utility function to explore the effects of household consumption through a minor absolute shrinkage and selection operator (LASSO) regression model. Lasso regression belongs to machine learning, which reduces the overfitting measurement error, compared to the ordinary multiple regression (29). The equation of household consumption and health education is as follows:


(1) $$\begin{array}{r} Y_{i}=\alpha +\alpha_{1}X_{CIi}+\alpha_{2}X_{2i}+\varepsilon_{i} \end{array}$$

(2) $$lasso=argmin(\sum {\left({\hat{Y}}_{i}-Y_{i}\right)}^{2}+\delta \sum \left|\alpha \right|$$

Where *X*_*CIi*_ (i = 1… i) are the independent variables of household consumption for individual i, α_1_ denotes the coefficient of health education. Because the index of household consumption is continuous, we used ordinary least square lasso regression.

### Consumption Relate to Health Education Inequality

**Figure 1:**
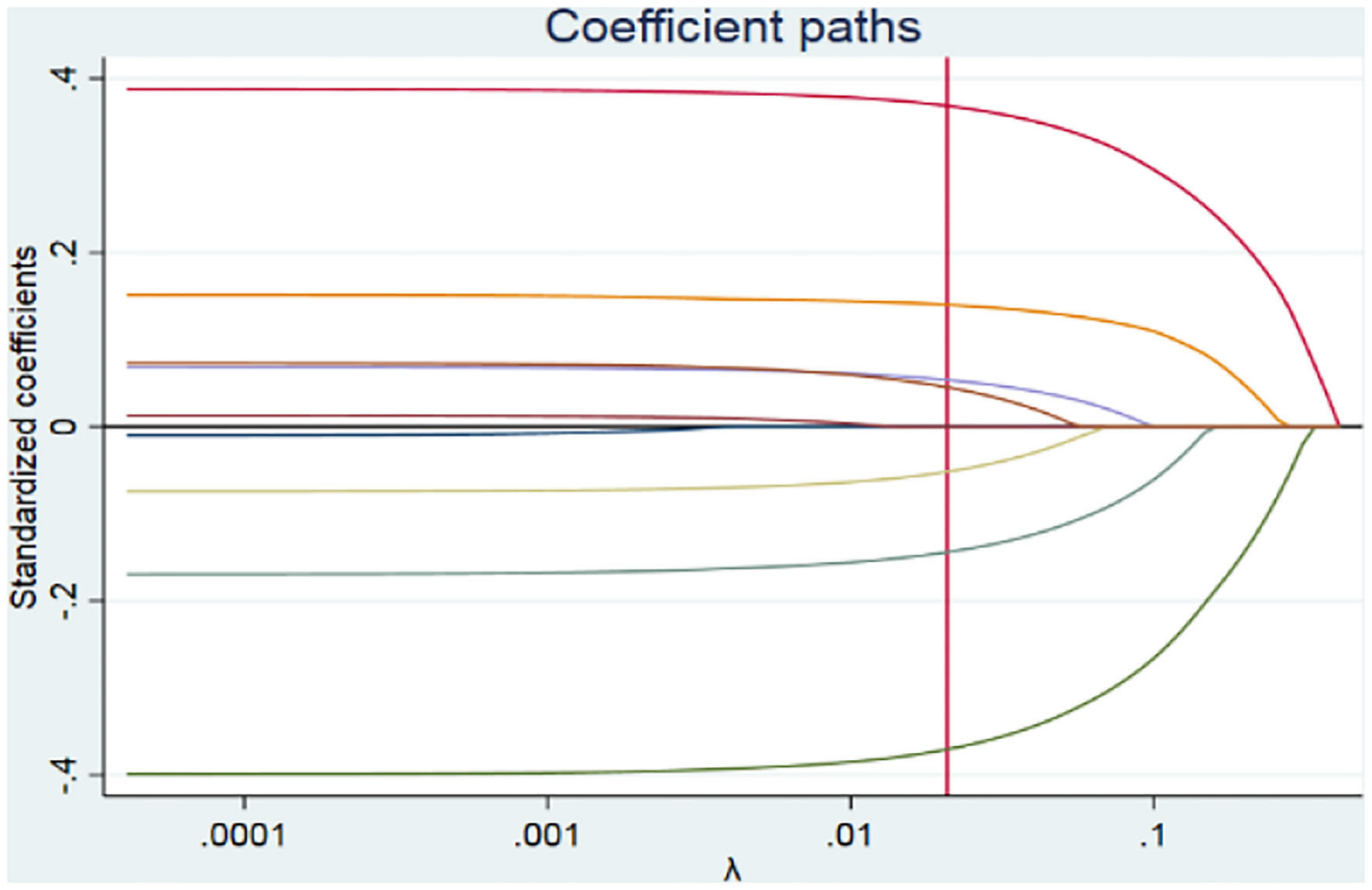
Lasso regression coefficient paths.

We analyze health education Inequality that employed a conventional method-calculation of concentration index (CI). The CI was initially proposed by Wagstaff et al. (30), which quantifies the degree of socioeconomic-related health inequality. The concentration index is defined as a concentration curve ranging from−1 to 1 (Figure 1). A concentration curve equal to the line of equality^3^ indicates that health education endowment is equal. If the curve is below the equality line, health education is concentrated among those with very low incomes. However, if the curve is above the equality line it means that health education is a focus among those with a high income. It is noteworthy that the distance between the concentration curve and the line of equality indicates greater health education inequality (31), The CI (concentration index) equation is as follows:


(3) $$\begin{array}{r} CI=\frac{2}{Y_{M}}COV\left(Y_{i,}R_{i}\right) \end{array}$$

Equation (3) defines the individual the degree of health education, indicating the mean of health education, and represents the quantile ranks of individual household consumption distribution. However, given the individual demand difference for health education, the CI (concentration index) only reflects the degree of inequality of health education.

Therefore, we also assess the HI (horizontal index) to evaluate the demand difference in health education among socioeconomic difference groups (32). A horizontal index (HI) of greater than zero indicates more needs for health education among those with a higher socioeconomic status, and a horizontal index (HI) below zero indicates more need for health education among low-income groups (33). The HI (horizontal index) was evaluated by subtracting the total need factor contributions from the CI (the need factors represented gender, education years, health status, or age). The equation for the HI (horizontal index) was as follows:


(4) $$HI=W_{ch}-\sum \left(\frac{\beta_{J}^{W}X_{J}}{Y_{M}}\right)C_{J}-\sum \left(\frac{\delta_{K}^{W}Z_{K}}{Y_{M}}\right)\;C_{K}-\frac{\theta C_{\varepsilon }}{Y_{M}}$$

Where *C*_*J*_ and *C*_*K*_ are the concentration indices for need factor *X*_*J*_ and none-need factor *Z*_*J*_, respectively. The partial effects of need factor and none-need factor were defined as $\beta_{J}^{W}$ and $\delta_{K}^{W}$. The $\sum \left(\frac{\beta_{J}^{W}X_{J}}{Y_{M}}\right)C_{J}$ represent the contributions of need factor, and the $\sum \left(\frac{\delta_{K}^{W}Z_{K}}{Y_{M}}\right)\;C_{K}$ present none-need factor contributions. The final section in equal (4) means the remaining error (34).

## Results

### All Variables Statistics

Table 1 displays all variable statistics among the 7,715 samples from the studies. Approximately one-third of the participants were aged from 21 to 45 years and 46 to 55 years. The average age was 35 years old, and the age square is 1369.67. More than half of the participants were married. Approximately one half of the participants were male (51.91%), and 48.09% of participants are female. In terms of education level, the average degree of education was junior school, and one-third of the participants were employees. Retired participants accounted for 8.7% and most participants were employed in the service industry. Nearly half of the participants were from urban households. More than one-third of the participants were covered by private commodity healthcare insurance.

**Table 1:** Socioeconomic statistic.

**Variables**	**Definitions**	**Obs**.	**Mean**	**Std. dev**.	**Min**	**Max**
Log household consumption	Household consumption points	7,715	0.33	0.19	0.00	1.00
Health education	Health education score	7,715	12.17	7.72	0.00	32.00
Private medical insurance	1 = purchasing 0 = none-purchasing	7,715	0.37	0.25	0.00	1.00
Gender	Male = 1; female = 0	7,715	0.51	0.49	0.00	1.00
Ages	Age squared	7,715	1369.67	836.20	324.00	9801.00
Education years	Education years	7,715	9.86	7.67	0.00	20.00
Married	Married = 1 Unmarried = 0	7,715	0.59	0.37	0.00	1.00
Identification residence	Rural = 1; Urban = 0	7,715	0.44	0.36	0.00	1.00
Residence status	Rental = 0; government house = 1; commodity = 2	7,715	0.57	0.31	0.00	2.00
Family member	Family members	7,715	3.07	1.17	1.00	10.00
Monthly income	1st quantile; 2nd quantile; 3rd quanttile; ndt quantile	7,715	2.57	1.19	1.00	10.00
Annual income	1st quantile; 2nd quantile; 3rd quanttile; 4nd quantile	7,715	3.17	1.44	1.00	6.00
Work status	Unemployment = 1; self-employment = 2; Retire = 3; Employee = 4	7,715	3.71	1.74	1.00	4.00
Occupation status	Agriculture = 1; Manufacturing = 2; Service Industry = 3	7,715	2.37	0.44	1.00	3.00

In terms of health education and household consumption, the average score for participant's health education was 12.17 points and the standard error was 7.72. The results indicate primary responders do not have enough health education, and concerns about health education inequality continue. The mean score of household consumption was 0.33; indicating that most participants had a low-level of consumption, with more preference for saving.

### Lasso Regression of Health Education and House Consumptions

We first explored the relationship between household consumption and other contributing variables to health education through Lasso regression, as shown in Table 2. Table 2 compares Ordinary Least Squares regression (OLS) with Post Double Lasso regression results. Post Double Lasso regression relieves the measurement errors caused by omitted variables and accurately identifies the other contributing variables for health education (28, 35). The consistency of Lasso and OLS results indicates the robustness of the lasso regression results.

**Table 2:** Lasso Regression of the relationship between health education and household consumption.

**Dependent variable**	**OLS**	**Post double lasso**
Household consumption	0.3087*** (0.001)	0.3087***
Ages	−0.3494*** (0.007)	−0.3494***
Edu years	0.131*** (0.002)	0.131***
Annual income	0.0601*** (0.0002)	0.0601***
Identification residence	−0.1271*** (0.0182)	−0.1271***
Private Medical Insurance	−0.0242*** (0.001)	−0.0242***
Health status	0.0437*** (0.0001)	0.0437***
_cons	4.27*** (0.037)	N
Cities control	Yes	Yes
Community control	Yes	Yes
Adjust R^2^	0.25***	N
CV fold	N	10
selected lambda	N	0.0208
Number of observations	7,715	7,715

Standard errors in parentheses, * p <0.10, ** p <0.05, *** p <0.01; The values in brackets in the cluster individual error. All results fix community level and city level.

The resulting report on household consumption showed significant positive effects on health education (coef = 0.3087, *P* < 0.001), indicating that the household consumption improves per unit. Health education enhances 30.87%. In terms of other contributing variables, such as age, education years; monthly income; residence; private medical insurance; and health status are all statistically different at the 1% significance level. However, age has a negative association with health education (coef = −0.3494, *P* < 0.001), other variables for the positive effects on health education suggest that with the age increasing, there is less access to enhanced health education.

Figure 1 shows the lasso regression results of coefficient paths, the selected lambda is 0.0208. As the penalty increases, the standardized coefficients are eventually compressed at zero (36). At λ of 0.0208, seven coefficients are not equal to zero. Therefore, there are seven contributing variables to health education. The results also imply that there is a health education disparity between those in rural and urban residential areas. Furthermore, participants who purchased private medical health insurance tend to have higher health education than those with basic healthcare insurance. In other words, concerns about household consumption causing health education inequality are serious across China.

### Consumption-Related Health Education Inequality

**Figure 2:**
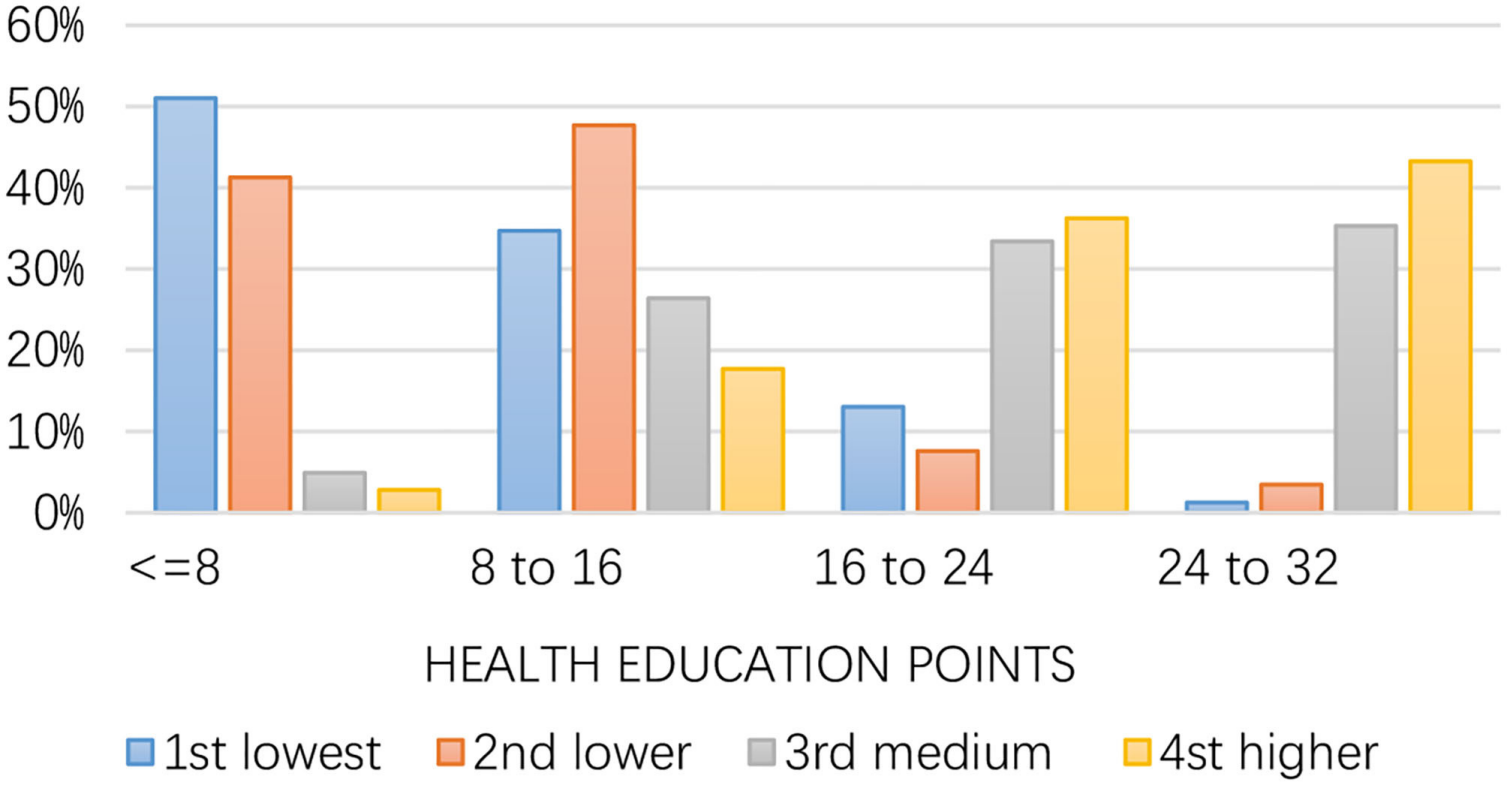
Health education, by consumption groups.

We further considered the heterogeneous degree of health education inequality across different consumption groups (defined by the household consumption point's quantile). We first performed a visual analysis of consumption-related COVID-19 health education inequality. Figure 2 indicates that the lowest health education score was in the lowest consumption group. The higher the health education score, the higher the consumption group. Approximately half of the participants with the first lowest consumption had health education points below the eighth score (53%). The other first lowest consumption participants had points that ranged from 8 to 16 (35%). On the contrary, the highest health education score parts from 24 to 32 are mostly third medium consumption and fourth higher consumption participants, respectively (38; 46%). Those results are consistent with Qian and Fan (37) and lasso regression, the result implies the existence of consumption-related inequality in health education.

**Figure 3:**
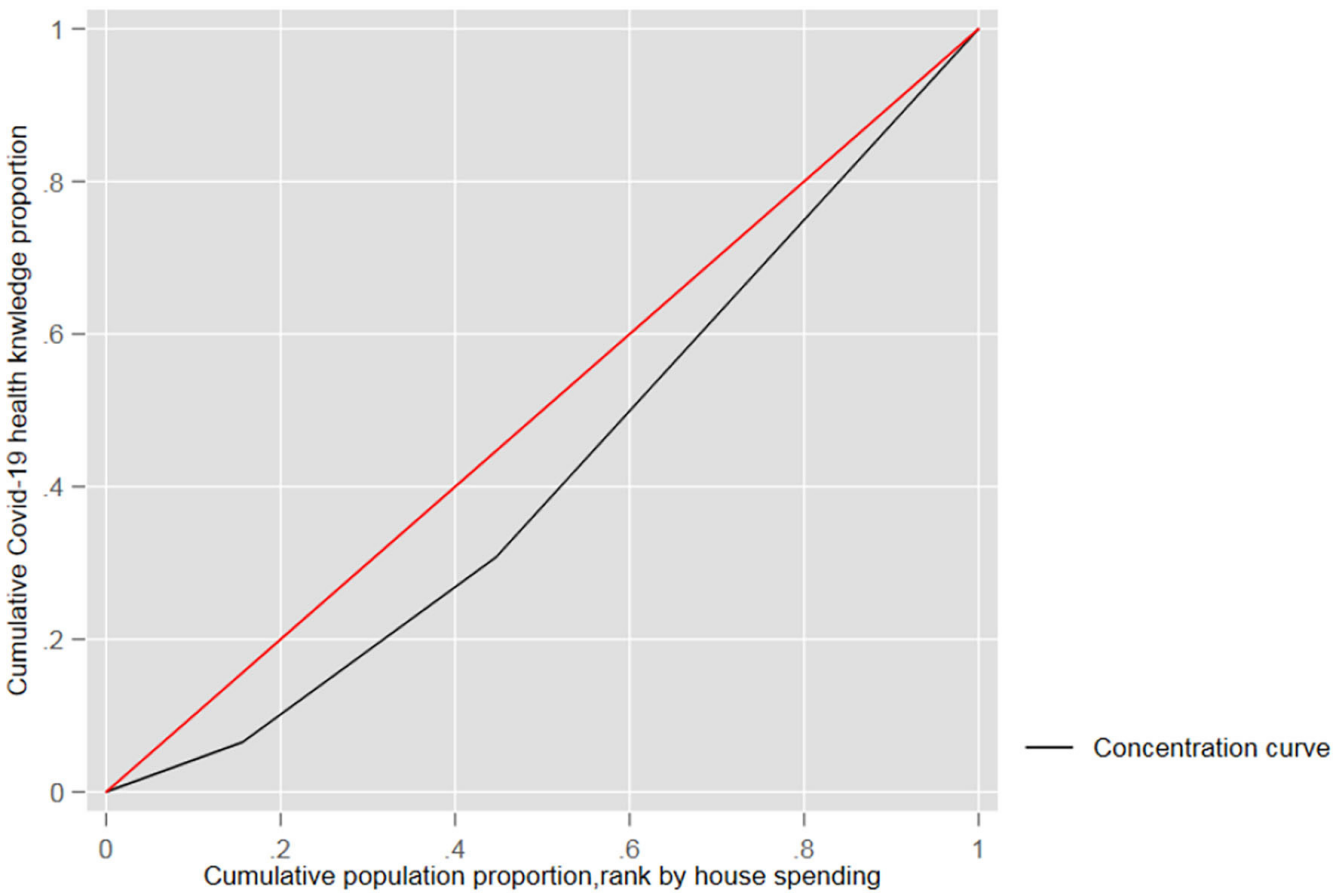
Consumption-related inequality in health education.

Figure 3 shows the concentration curves for consumption-related health education inequality during COVID-19. The visual results indicate a significant inequality in health education against the lowest consumption, indicating the lowest consumption probably has the lowest income according to the income consumption curve (38, 39). Therefore, participants with lower consumption have lower access possible to improve health education and prevent knowledge during the pandemic.

According to the health education concentrated curve, health education is concentrated among responders with higher consumption, not responders with the lowest consumption (CI: 0.0321; *P* < 0.0001). The concentration index (CI) means that the inequality among high-low income was 0.0321, and the 95% confidence interval range was 0.005 to 0.072.

To summarize, the consumption concentrate result shows that only participants with higher consumption have the possibility of enhancing health education. The results of CI indicate pro-rich inequality in health education. The empirical results also indicate that improved income can mitigate health education inequality. Kaidi and Mensi (40) also suggests that in addition to economic development, an excellent income distribution political system can also improve education inequality.

Apart from the consumption-related health inequality assessment, we also analyzed the income CI and HI inequality index in Table 3. Considering the assumption about income-consumption theory, heterogeneous individual incomes have different influences on health education. In other words, according to income-consumption theory, income may affect health education inequality (41).

**Table 3:** CI and HI of inequality in health education by income.

	**CI**	**HI**	**Mean ±SD**
All	0.0321***	0.0416***	
Household consumption (lowest)			0.23 ± 0.15
Household consumption (lower)			0.38 ± 0.08
Household consumption (Medium)			0.32 ± 0.22
Household consumption (Higher)			0.22 ± 0.12
Annual income (low)	0.0092***	0.0171***	
Household consumption (lowest)			0.14 ± 0.02
Household consumption (lower)			0.17 ± 0.12
Household consumption (Medium)			0.64 ± 0.42
Household consumption (Higher)			0.74 ± 0.52
Annual income (middle)	0.0107***	0.298***	
Household consumption (lowest)			0.54 ± 0.41
Household consumption (lower)			0.57 ± 0.52
Household consumption (Medium)			0.71 ± 0.57
Household consumption (Higher)			0.69 ± 0.62
Annual income (Higher)	0.0171***	0.169***	
Household consumption (lowest)			0.64 ± 0.47
Household consumption (lower)			0.71 ± 0.62
Household consumption (Medium)			0.81 ± 0.72
Household consumption (Higher)			0.84 ± 0.65

Annual Income divided as income tertiles, with low (1st tertile), middle (2nd tertile), and high (3rd tertile); Household consumption defined as consumption quantile, with low (1 st quantile), middle (2nd quantile), and high (3rd quantile); CI Concentration Index, HI horizontal index. *P <0.10, **p <0.05, ***p <0.01; All results fix community and city level.

### Heterogeneous Effects of Income Perspective

We estimated income CI and HI index to re-estimate our results in Table 3. The empirical results show that the annual income CI index and HI index for low-income groups are 0.0321 and 0.0416, respectively. In terms of wealth groups, the annual income CI index is equal to 0.0171, all results have statistical significance at 1%. The relationship between income and health education shows that health education concentrates on wealthier participants (higher income), indicating that participants with a higher income have higher health education (42).

Additionally, the heterogeneous effects of the annual income of China also were observed empirically. The low annual income HI index was 0.0171, indicating the lowest health education at COVID-19 among the lowest consumption. For higher-annual income groups, the HI index was 0.169, implying higher health education is consistent in higher consumption groups. All indices were statistically both significant and positive, which means health education concentrated on higher household consumption and income groups.

### Decomposition of Consumption-Related Health Education Inequality

From the perspective of household consumption and contributing variables for health education, the decomposition results are reported in Table 4. These results show that household consumption accounted for 37.2% of the contribution to health education inequality, indicating consumption is the major contributor to inequality in health education. Annual income is the second-largest contribution to health education inequalities (27.1%), the results are consistent with Table 3. In terms of other contributing variables, education years, private healthcare insurance, health status, and identification citizenship all contribute to health education inequality, the contributing coefficients are 17.8, 3.8, 9.7, and 6.4%, respectively.

**Table 4:** Decomposition of health education inequality.

	**CI**	**Contribution**	**Elasticity**
Household consumption	0.0321	0.372	0.143
Annual income	0.0272	0.271	0.127
Ages	−0.0270	−0.002	0.106
Education years	0.0181	0.178	0.099
Identification citizenship	−0.0073	0.064	0.035
Private medical insurance	−0.0101	0.038	0.007
Health status	0.0065	0.097	0.014
Cities	−0.027	−0.027	0.217
Community	0.0279	0.007	0.266
Total		100%	

CI Concentration Index of factor; * P <0.10, ** p <0.05, *** p <0.01; Contribution is defined as the contribution of each factor to the total inequality.

For the elasticity coefficients, the results show that with household consumption increases of 1% the health education inequality extends to a 0.143% ratio. The deterioration of income distribution can make health education inequality enhance by a 0.127% ratio. All the results focus on variables that can affect health education inequality.

The contributing factors selected by Lasso machine regression, lead to a residual value of zero, indicating that the regression improves the measurement error caused by omitting factors (43).

**Figure 4:**
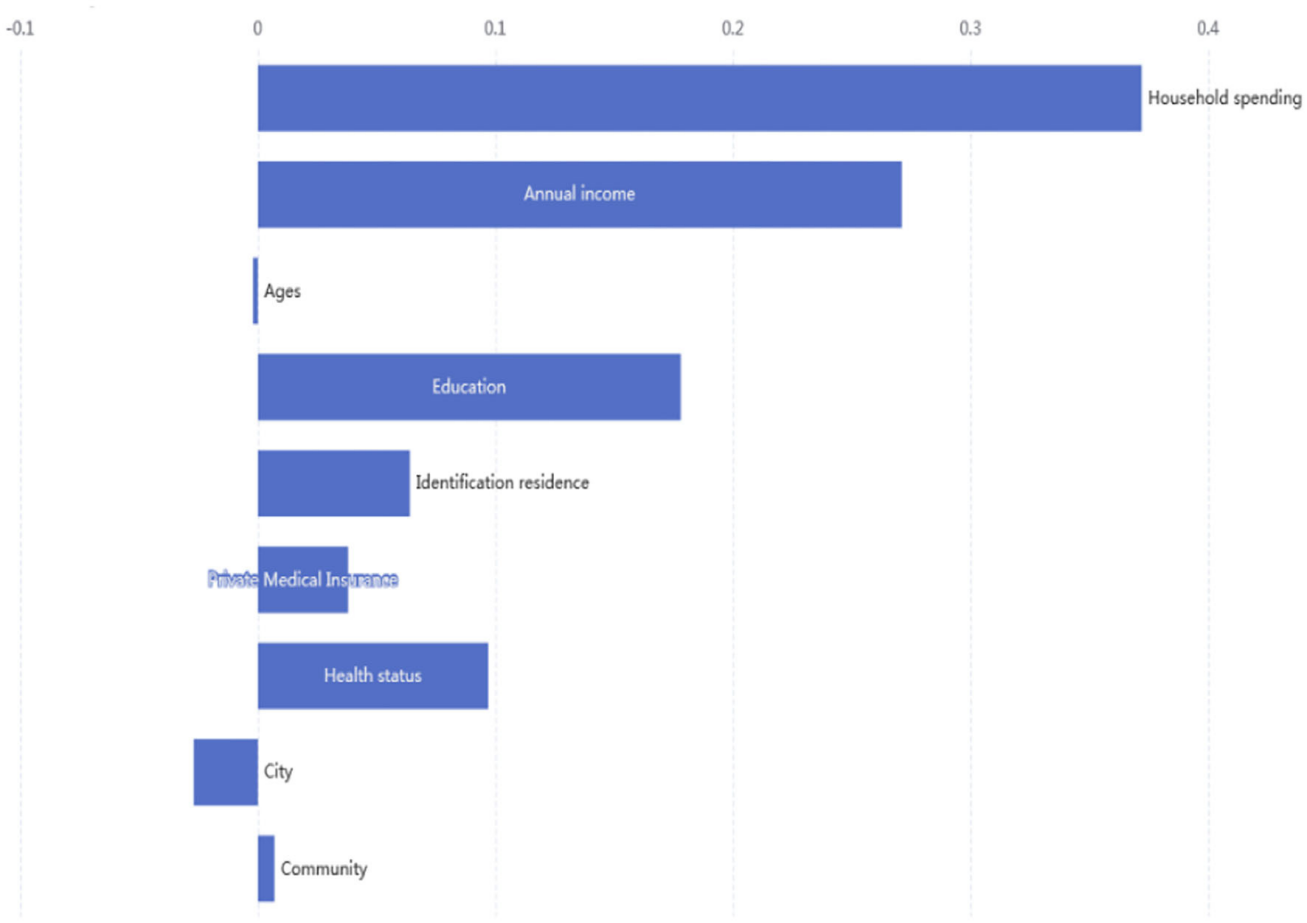
Contributions of socioeconomic to inequality in health education.

Figure 4 shows the contributions of COVID-19 health education inequality in distinguishing socioeconomic factors. The summarized contributions of household consumption to inequality were 37.2%, and annual income accounted for 27.1% of health education inequality. However, cities reduced inequality in health education. Moreover, individuals with higher education and health status contribute to inequality by 17.8 and 9.7%, respectively. At the same time, a better atmosphere among urban residents leads to a quicker gain in health education, as healthcare service increases inequality by only 0.7%.

## Discussion

This study aims to investigate the degree of household consumption related to inequality in health education during COVID-19 and other contributing variable effects for health education inequality through a cross-sectional survey during the pandemic across China. These results reveal that the health education in low consumption groups was lower than those for the high consumption preference group. In other words, the majority of low economic status individuals undertook lower health education. These findings are consistent with previous studies on health inequalities (44, 45), which revealed that wealthy groups had better health education than low-income groups. The CI and HI health education were 0.0321 and 0.0416, respectively, indicating that health education is concentrated among high-income populations. The possible explanation for this is that with higher consumption preferences, individuals with a high income have more opportunities to obtain health education.

The Decomposition of the concentration index revealed that income level, education, and identification residence are the most significant drivers of inequality across China. These findings suggest that with higher education, individuals undertake better health education about COVID-19. This finding is supported by other literature (19, 46, 47). The possible reason for this is that education is a symbol of human capital, closely related to family culture capital. They have more awareness of how to prevent COVID-19 by obtaining health education. The Chinese public healthcare system still presents several challenges, such as health coverage and identification of residence inequality (48). Notably, the healthcare cost does not cover people on a low income for all costs, so disparities in healthcare worsen consumption-related inequalities.

Consequently, access to healthcare has not improved among people with a low income, and healthcare expenses have restricted consumption preferences during COVID-19. No other studies to date have considered the rural-urban residence concentration index in China. Our results found that health education along with rural and urban identification also contributes to inequalities in household consumption preferences. The possible reason for this is that the “New Rural Cooperative Medical Scheme” healthcare system is inadequate in terms of coverage and access to health education in rural areas. Therefore, rural residential populations do not have enough extra expenditure for items other than medical costs. Compared to rural healthcare, most citizens purchase private health medical insurance, indicating higher quality healthcare and comprehensive knowledge-spreading channels. Additionally, urban residents have more access to health education about COVID-19, whereas it is a lot more challenging for rural residents to access the latest health education and healthcare.

## Limitations

This is the first study to evaluate household consumption inequality toward health education across China during the COVID-19 pandemic. These findings may contribute to local governments making economic recovery policies in the future. However, this study has some limitations that should be considered. We based these results on a self-reported sample to analyze inequality, which might cause some measurement bias. Second, we cannot identify causal effects between household consumption and health education by cross-section survey. Therefore, future research might adopt a panel dataset to address the endogenous issue. Finally, we only analyze the relevant relation, not considering a casual relationship. It is noteworthy that the results are based on lasso regression to reduce endogenous issues caused by omitted variables. That said, future research should pay attention to the instrumental variables to assess whether there is a relationship between COVID-19 health education and household consumption.

## Conclusions

This study investigated the degree of consumption-related inequality in health education during COVID-19, based on a machine learning method. It aimed to establish the main socioeconomic characteristics that contribute to inequality across China. These results have important policy implications. Firstly, the government should improve income to mitigate the negative economic impact of COVID-19 among people with a low income. Secondly, the government should also adopt intervention programs and strategies, such as subsidy policies to promote health education among people with a low income to reduce inequality. Third, we have observed the effects of private health insurance on COVID-19 health education. Therefore, the government should gradually enhance public health insurance coverage to reduce consumption anxiety attitudes during pandemics, which would help improve consumption-related health education inequalities.

## Data Availability Statement

The raw data supporting the conclusions of this article will be made available by the authors, without undue reservation.

## Author Contributions

JY: formal analysis, methodology, visualization, and writing–review and editing. JZ: revise. ZL: data curation and writing–original draft. All authors contributed to the article and approved the submitted version.

## Funding

This work was funded by National Natural Science Foundation of China (Grant No. 71773068) and Shanghai University of Finance and Economics Postgraduate Innovation Fund (Grant No. 20210821).

## Conflict of Interest

The authors declare that the research was conducted in the absence of any commercial or financial relationships that could be construed as a potential conflict of interest.

## Publisher's Note

All claims expressed in this article are solely those of the authors and do not necessarily represent those of their affiliated organizations, or those of the publisher, the editors and the reviewers. Any product that may be evaluated in this article, or claim that may be made by its manufacturer, is not guaranteed or endorsed by the publisher.

## Footnotes

^1^The Cronbach equal 0.895 analyzed in Statistical Package for the Social Sciences (SPSS).

^2^Data source: http://charls.pku.edu.cn/pages/data/111/zh-cn.html.

^3^The 45-degree line.
